# Quantum-Dot Light-Emitting Diodes with Nitrogen-Doped Carbon Nanodot Hole Transport and Electronic Energy Transfer Layer

**DOI:** 10.1038/srep46422

**Published:** 2017-04-12

**Authors:** Young Ran Park, Hu Young Jeong, Young Soo Seo, Won Kook Choi, Young Joon Hong

**Affiliations:** 1 Graphene Research Institute, Sejong University, Gwangjin-gu, Seoul 05006, Republic of Korea; 2 UNIST Central Research Facilities (UCRF), UNIST, Ulsan 44919, Republic of Korea; 3 Department of Nanotechnology and Advanced Materials Engineering, Sejong University, Gwangjin-gu, Seoul 05006, Republic of Korea; 4 Materials and Life Science Research Division, KIST, Seongbuk-gu, Seoul 02792, Republic of Korea

## Abstract

Electroluminescence efficiency is crucial for the application of quantum-dot light-emitting diodes (QD-LEDs) in practical devices. We demonstrate that nitrogen-doped carbon nanodot (N-CD) interlayer improves electrical and luminescent properties of QD-LEDs. The N-CDs were prepared by solution-based bottom up synthesis and were inserted as a hole transport layer (HTL) between other multilayer HTL heterojunction and the red-QD layer. The QD-LEDs with N-CD interlayer represented superior electrical rectification and electroluminescent efficiency than those without the N-CD interlayer. The insertion of N-CD layer was found to provoke the Förster resonance energy transfer (FRET) from N-CD to QD layer, as confirmed by time-integrated and -resolved photoluminescence spectroscopy. Moreover, hole-only devices (HODs) with N-CD interlayer presented high hole transport capability, and ultraviolet photoelectron spectroscopy also revealed that the N-CD interlayer reduced the highest hole barrier height. Thus, more balanced carrier injection with sufficient hole carrier transport feasibly lead to the superior electrical and electroluminescent properties of the QD-LEDs with N-CD interlayer. We further studied effect of N-CD interlayer thickness on electrical and luminescent performances for high-brightness QD-LEDs. The ability of the N-CD interlayer to improve both the electrical and luminescent characteristics of the QD-LEDs would be readily exploited as an emerging photoactive material for high-efficiency optoelectronic devices.

Quantum-dot light-emitting diodes (QD-LEDs) have recently attracted a great deal of attention for their diverse optoelectronic device applications^1^,^2^, owing to excellent physical properties of QDs^3^ (*i.e*., bandgap tunability over wide spectral range, narrow emission bandwidth, high quantum efficiency, *etc*.). Despite such advantages, the electroluminescence (EL) efficiency of QD-LEDs still remains a critical issue for use in indoor light illumination or thin film display applications^4^. It is well known that the EL efficiency of QD-LEDs is essentially determined by two major factors: (i) *p*-type conductivity of hole injection and transport layers (HILs and HTLs)^5^ and (ii) luminescence efficiency of the QD layer^6^. As for the approaches, the enhancement of *p*-type conductivity has been conducted by reducing the height of the highest hole barrier in multilayer HTL heterojunctions^7^ or adjusting a series of the highest occupied molecular orbital (HOMO) levels of HTLs by inserting a new layer^7^,^8^,^9^,^10^ or adopting the inverted QD-LED structure^11^,^12^; the luminescence performance of QDs has also been improved by employing the surface passivation ligands, electron overflow barrier^13^, or conjugating exciton- (or carrier-) injection layer to the QD layer^13^,^14^,^15^. To date, these approaches have been maturely developed as separate factors for high-efficiency QD-LEDs^4^,^6^,^16^. However, it is suggested that an ideal way to improving the EL efficiency is to make use of a layer (i) that can improve the *p*-type conductivity in the QD-LED structure and (ii) can simultaneously enhance the radiative recombination of QD layer.

Graphene is a one (or few)-atom-thick graphitic layer with a unique bi-conical zero bandgap electronic structure, and importantly, the bandgap could have been modulated by changing the size, shape and edge states of graphene via quantum confinement and/or edge auxochromic effect^17^,^18^,^19^. Moreover, the electrical conductivity as well as the work function of graphene is amphoterically tunable by chemical and physical doping^20^,^21^,^22^. Due to the ease of the electronic structure and optical property manipulation, the graphene nanostructures (*i.e*., graphene nanodot, graphene quantum dot, carbon nanodot, *etc*.) have been exploited as hole (or electron) transport layer or lumophore layer for optoelectronic devices^23^,^24^,^25^. Specifically, the carbon nanodots (CDs), fabricated by bottom-up solution synthesis, also have been demonstrated as an emerging photoactive layer with the capacity of ultraviolet (UV)–visible fluorescence^26^,^27^, luminescence up-conversion^28^,^29^, and hot carrier generation^30^, all of which are endowed by the aforementioned physical properties. Moreover, the bottom-up synthesis of CDs enables atomic and/or functional group doping with high dopant solubility because the low temperature synthesis causes high density vacancy or imperfections where the dopants can be incorporated^31^,^32^,^33^,^34^. Thus, the CDs deserve to be utilized as a surrogate layer for many optoelectronic devices.

Herein, we report the enhanced electrical and EL performance of solution-processed red QD-LEDs by insertion of nitrogen-doped CD (N-CD) layer. The QD-LEDs and/or hole-only devices (HODs) with/without N-CD interlayer were comparatively characterized by electrical measurement and electronic structure analyses. FRET (Förster resonance energy transfer)-assisted luminescence enhancement and *p*-type conduction enhancement with reduced hole barrier height, given by the insertion of N-CD layer between the multilayer HTL and QD layer, were validated by photoluminescence (PL) and ultraviolet photoelectron spectroscopies (UPS), respectively. The synthesis and diverse spectroscopic characteristics of the N-CD layer were also demonstrated.

## Results and Discussion

### Nitrogen-Doped Carbon Nanodots

**Figure 1:**
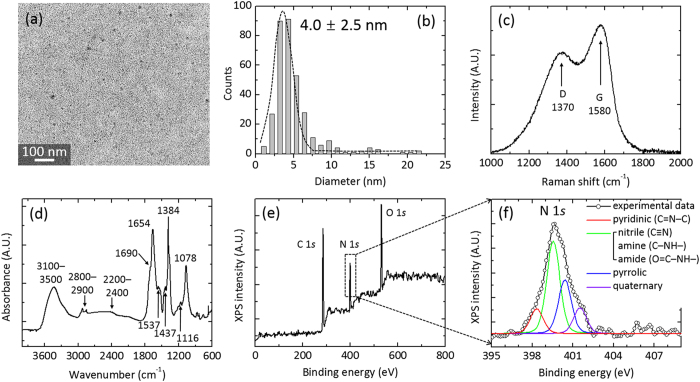
Electron microscopic and spectroscopic analyses of N-CDs. (**a**) TEM image and (**b**) diameter histogram of the N-CDs. (**c**) Raman, (**d**) FT-IR, (**e**) XPS, and (**f**) peak-fitted N 1 *s* XPS spectra of N-CD layer. The peak fitting was performed by peak de-convolution using symmetric Voigt functions.

The N-CDs were synthesized via solution-based cross-linking reaction using ethanolamine. The detailed synthesis process is described in the Methods section. The morphology and diameter distribution of N-CDs were investigated by high-resolution transmission electron microscopy (HR-TEM). Figure 1a is a low-magnification TEM image of N-CDs dispersed onto the carbon-coated TEM grid, presenting small nanoparticle morphology with homogeneous size distribution. The diameter distribution of N-CDs was measured to be a mean value ± standard deviation of 4.0 ± 2.5 nm through HR-TEM analysis (Fig. 1b). Specifically, the crystallinity of N-CDs was clearly observed via HR-TEM inspection (Figure S11b, Supplementary Information).

The molecular structure and chemical state of the N-CD were characterized using Raman, Fourier transform infrared (FT-IR), and x-ray photoelectron spectroscopies (XPS). The Raman spectrum of the N-CD in Fig. 1c shows two dominant peaks centered at 1370 and 1580 cm^−1^, which are assigned to the D band arising from vibrations of *sp*^3^-hybridized carbon atoms with dangling bonds or disordered carbon atoms and the G band associated with the E_2g_ mode of graphite due to the vibration of *sp*^2^-bonded carbon atoms in a two dimensional hexagonal lattice, respectively^35^,^36^. The peak intensity ratio of the D and G bands (I_D_/I_G_) was measured at 0.89, and the widths of the D and G peaks were measured to be 230 and 110 cm^−1^, respectively. The high intensity of the D peak and broad width of the D and G peak imply the presence of high-density defects (or higher disorder)^35^ and/or high-concentration dopants on the defect sites of the CD^37^,^38^. The I_D_/I_G_ value and width of D band in this study is comparable to those of CD or graphene-QD reported elsewhere^32^,^36^.

Figure 1d displays FT-IR spectrum of the N-CD. The FT-IR absorbance peaks centered at 1116 and 1654 cm^−1^ are attributable to the presence of the *sp*^3^-hybridized C–C bonds and *sp*^2^-hybridized C=C bonds of graphitic carbon, respectively. Because of N-doping, a broad band at 2200–2400 cm^−1^ can be assigned to the stretching of C≡N (*sp*^1^) bonds^33^, then the peaks at 1116 and 1654 cm^−1^ are partially attributable to the asymmetric stretching of *sp*^3^ C–N^39^ and *sp*^2^ C=N bonds^34^, respectively. The broad absorbance band at 3100–3500 cm^−1^ is an ensemble of vicinal peaks related to intermolecular interactions via H-bridges in the N-CD^40^; specifically, the absorption band in wavenumber range of 3100–3300 cm^−1^ is attributable to symmetric stretching of NH, and that of 3300–3500 cm^−1^ to the anti-symmetric stretching of NH. In addition, peaks at 1116, 1537, and 1384 (and 1437) cm^−1^ are ascribed to the C–N bonds stretching in the primary amines, in-plane bending of N–H in the secondary amine, and in-plane bending of N–H in the tertiary amine, respectively^39^,^41^. The absorption shoulder at 1690 cm^−1^ is assigned to the amide carbonyl (C=O) stretching. The observation of several absorbance peaks related to C=O and NH indicates the existence of the primary amino group and secondary amino functional group [O=C–NH–] in the N-CDs^42^, signifying that N atoms were incorporated as various chemical forms into the CDs via the solution-based growth process. Meanwhile, two absorbance peaks in the wavenumber range of 2800–2900 cm^−1^ are ascribed to the stretching vibration of the –C–CH_3_ and ≡CH bonds, and the peak at 1437 cm^−1^ is attributed to the asymmetric bending of CH_2_^34^. The peak at 1078 cm^−1^ is tentatively assigned to either the C–O stretching vibrations of epoxy or alkoxy groups or the symmetrical stretching vibration of the aryl ether (C–O–C) as an oxygen substitute in the N-CD^43^. The FT-IR analysis implies that the N-CDs were well synthesized by a polymerization process via cross-linking reactions between ethanolamine and its oxidative products.

The elemental composition and chemical bonds of the N-CD were investigated using XPS. In Fig. 1e, the XPS data obtained in wide scan range exhibits three dominant peaks centered at binding energies of 284.5, 400.1, and 532.1 eV, which correspond to C 1 *s*, N 1 *s*, and O 1 *s* peaks, respectively. The elemental content of the N-CDs was estimated to be C 64 at.%, N 17 at.%, and O 19 at.% by measuring the intensity ratio of the integrated peaks, implying that substantial amounts of N, O, and N-related functional groups were incorporated into the CD via the solution-based carbonization process^38^,^44^. Hence, our product must be the oxygen-rich N-CD. However, we note that the N content (17%) in our N-CD is much greater than that of N-doped graphene nanostructures reported elsewhere (0.3–5.6%)^45^,^46^.

The chemical form of the nitrogen species in the N-CD was further characterized by multiple-peak fitting of the N 1 *s* peak using symmetric Voigt functions. The N 1 *s* peak was de-convoluted into four peaks, as shown in Fig. 1f: the peak at 398.3 eV (13.7 at.% calculated among N-relevant peaks) is attributed to the existence of pyridinic N (C=N–C as *sp*^2^-hybridized form); the most dominant peak at 399.5 eV (46.9 at.%) is due to abundant presence of amine (–NH–, –NH_2_), carbonitrile (–C≡N, *sp*^1^-states of N), or amide functional groups (–N–C=O–); the peak at 400.3 eV (27.4 at.%) is tentatively resulted from pyrrolic N (N-incorporated 5-membered heterocyclic ring); the XPS peak at 401.4 eV (12.0 at.%) is ascribed to quaternary N (*sp*^2^-hybridized N bonded with three *sp*^2^-hybridized C neighbors). XPS analysis shows that the nitrogen was incorporated as the atomic impurity (*i.e*., pyridinic, pyrrolic, and quaternary N)^47^,^48^ and N-related functional groups^25^,^42^,^46^. Detailed chemical forms of atomic N dopant are schematically depicted in Supplementary Figure S2. The multiple-peak fitting analyses for C 1 *s* and O 1 *s* are also provided in the Supplementary Information Section (see Supplementary Figure S3).

### Electrical and Electroluminescent Performances of QD-LEDs with/without N-CDs Interlayer

**Figure 2:**
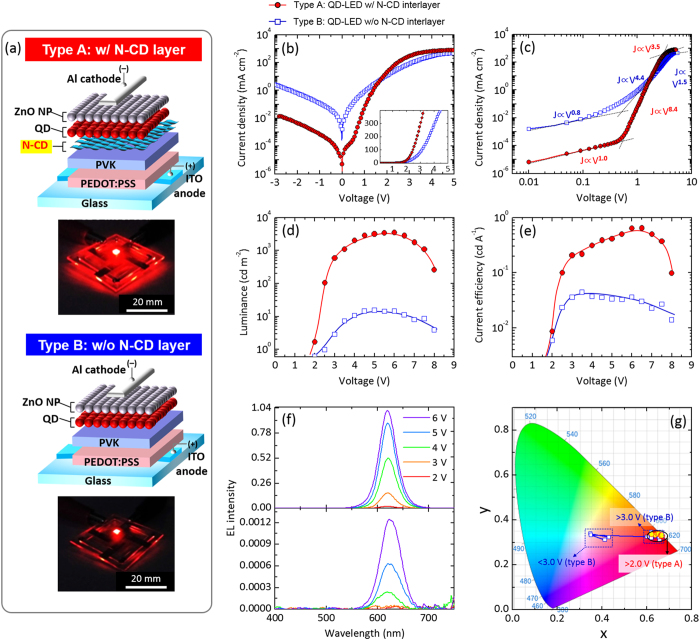
QD-LEDs with/without N-CD interlayer. (**a**) Schematic illustrations depicting the structures of solution-processed QD-LEDs. The photographs of red emission from both types of QD-LEDs were taken at a driving voltage of 5 V under the same dark room and camera conditions. (**b**) *J*–*V* characteristic curves. Inset is plotted in a linear scale at forward applied voltages. (**c**) *J*–*V* characteristic curves plotted on double-logarithmic axes. (**d**) Luminance and (**e**) Current efficiency plotted as a function of applied bias voltage. (**f**) EL spectra of the N-CD-inserted QD-LED (upper panel) and the control QD-LED (lower panel) at diverse applied voltages. (**g**) CIE coordinates of EL emission colors measured at various applied bias voltages of 2.0–8.0 V.

Figure 2a schematically illustrates the structures of solution-processed QD-LEDs with/without N-CD interlayer. The QD-LEDs basically consisted of multilayer films of poly(3,4 ethylene-dioxythiophene) polystyrene sulfonate (PEDOT:PSS), poly(N–vinylcarbazole) (PVK), CdSe/CdZnS core/shell QD, and ZnO nanoparticle (NP), which were sequentially deposited on indium-tin oxide (ITO)-coated glass substrates by solution-based spin coating for the HIL, HTL, luminescence layer, and electron injection layer, respectively, for a control QD-LED (type B). The N-CD layer was inserted by spin coating between the PVK and QD layers for the type A QD-LED structure. No significant degradation or damage were observed after coating of each layer (Figure S1, Supplementary Information). The thicknesses of the PEDOT:PSS, PVK, N-CD, QD, and ZnO NP layers were ca. 30, 22, 4, 15, and 30 nm, respectively (Figures S9 and S10, Supplementary Information). The detailed methods of synthesis and device fabrication are described in Methods section. The 2.0 × 2.0-mm^2^ chips of the QD-LEDs (type A and B) exhibited uniform light emission over the entire chip area above the EL turn-on voltage, and the type A QD-LEDs especially showed higher brightness with respect to type B QD-LEDs under typical operating voltage conditions (insets of Fig. 2a).

The electrical and EL properties of QD-LEDs with/without N-CD interlayer were characterized and compared by measuring current density–voltage–luminance (*J*–*V*–*L*) curves and EL spectra, respectively. The *J*–*V* curves of Fig. 2b show that both types A and B QD-LEDs exhibited good electrical rectification with electrical thresholds of 2.2 and 2.9 V, respectively (see also inset of Fig. 2b). Dynamic resistance values of types A and B QD-LEDs above the threshold voltage (3.0 V) were estimated to be 59.9 and 192.4 Ω (for the LED area of 2.0 × 2.0 mm^2^), respectively; this indicated a much lower dynamic resistance for the type A QD-LEDs. Noticeably, type A QD-LEDs showed a steeper increase in terms of current density at forward applied voltages and much lower leakage current at reverse bias voltages in comparison with type B QD-LEDs, signifying superior diode characteristics of type A QD-LEDs with high rectification and low dynamic resistance. From double logarithmic plots in Fig. 2c, the *J*–*V* characteristics of both QD-LEDs are classified into three regimes of (i) an ohmic conduction (*J* ∝ *V*) at low applied voltage below 0.5 V, (ii) a trap-limited conduction (*J* ∝ *V*^*m*^, *m* > 2) for applied bias voltages ranging from 0.5 to 3 V, and (iii) a space-charge limited conduction (*J* ∝ *V*^*m*^, *m* ~ 2) at higher voltages over 3 V. In the ohmic conduction region, the current density of type A QD-LEDs was lower than that of type B by two orders of magnitude, indicating that the leakage current of type A QD-LEDs was significantly suppressed by inserting the N-CD layer; such a suppressed leakage current of type A QD-LEDs was also observed under a reverse bias condition. In the trap-limited conduction region, the slope of the *J*–*V* curve for the type A QD-LEDs was steeper than that of type B QD-LEDs, attributable to improved hole transport through the N-CD interlayer in type A QD-LEDs.

Noticeably, type A QD-LEDs typically exhibited several ten or hundred times higher luminance and luminous current efficiency than those of type B QD-LEDs above the electrical thresholds, as shown in Fig. 2d and e, respectively. The maximum luminance of types A and B QD-LEDs was 3500 and 20 cd m^−2^ for applied bias voltages of 6.0 and 5.0 V, respectively; the current efficiency of types A and B QD-LEDs represented the maximum values of 6.3 × 10^−1^ and ~4.4 × 10^−2^ cd A^−1^ at 6.0 and 3.5 V, respectively. Considering the *J*–*V* characteristics, such higher EL efficiency of type A QD-LEDs is thought to be strongly associated with enhanced hole transport through the N-CD layer.

The EL emission properties of the QD-LEDs were further investigated by EL spectroscopic analysis. Figure 2f shows the EL spectra of types A and B QD-LEDs, both of which present a dominant emission peak at 622 nm under high applied voltage conditions (>4.0 V), regardless of the N-CD insertion. This indicates that the EL emission mostly occurred by means of radiative electron–hole recombination at the QD layer in spite of the N-CD insertion. The EL intensity of type A QD-LEDs was typically two orders of magnitude higher than that of type B QD-LEDs at the same applied voltages (800 times at 6.0 V), and the EL emission of the type A QD-LEDs was strong enough to be observed with unaided eyes under normal indoor illumination conditions at a typical operating voltage of 3–6 V.

The EL emission color of both types of QD-LEDs was further characterized by determining the Commission International de l’Éclairage 1931 (CIE) coordinates at diverse bias voltages (see Fig. 2g). The EL color of the type A QD-LEDs at the applied bias voltage range of 2–8 V presented quite consistent CIE coordinates with a mean value ± standard deviation of (0.66, 0.33) ± (0.02, 0.01), corresponding to the highly pure red emission color. In contrast, the initial EL emission color of the type B QD-LEDs for an operating voltage of 2–3 V was an unstable whitish orange or orange–red with the CIE coordinates around (0.4, 0.35); as the applied bias voltage increased, the emission color shifted into the nearly pure red region of the CIE coordinate (0.66, 0.33). According to the PL spectra, type B QD-LEDs emitted a mixture of EL peaks centered at 622 nm and ~350–400 nm under the low applied bias voltage conditions, whose peaks are attributable to the radiative recombination at the QD layer and PVK film, respectively. The previous report of strong near-UV luminescence of the PVK film at an emission wavelength of 350–400 nm supports our argument^49^. Accordingly, it is surmised that the carrier charging at QD/PVK, because of insufficient hole injection in the type B QD-LEDs, was presumably responsible for radiative recombination not only at the QD but also at the PVK layer. In other words, the reduction in carrier charging (or sufficient supply of hole carriers) was made possible by the insertion of N-CD layer between PVK and QD layers (type A), resulting in highly pure red emission. Nevertheless, it is necessary to scrutinize the role of N-CD layer in the enhancement of electrical and EL performance of N-CD-inserted QD-LEDs.

### Optical Properties of QD/N-CD bilayer & Effect of N-CD Interlayer Thickness on Electrical and Electroluminescent Performances of QD-LEDs

**Figure 3:**
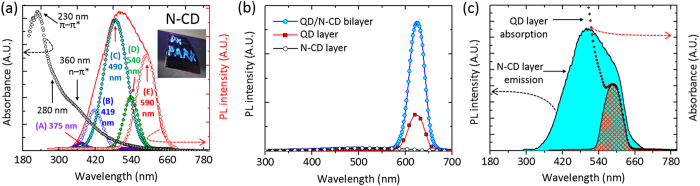
Optical properties of N-CD layer. (**a**) UV–visible absorption and PL spectra of N-CD layer. The PL band was de-convolved by multiple peak fitting. Inset is photograph of N-CD film lettered on Si substrate, taken under UV illumination. (**b**) PL spectra of N-CD, QD, and QD/N-CD layers. (**c**) PL spectrum of N-CD layer (black line) and UV–visible absorption spectrum of QD layer (red circles). The spectral overlap is marked with red checker.

The optical properties of both types of QD-LEDs were investigated by performing PL and UV–visible absorption spectroscopic analyses of the QD layer, N-CD layer, and QD/N-CD bilayer. Figure 3a shows the absorption (empty circles) and PL (red line) spectra of the N-CD layer at room temperature. The absorbance spectrum represents a broad band including three characteristic absorption transitions at 230, 280, and 360 nm with long tail above 450 nm. The absorption peak at 230 nm can be assigned to π-π* transition of the aromatic C=C bond, while the shoulder at 360 nm is caused by the n-π* transition of C=O bond^23^,^33^,^47^. Specifically, the absorption shoulder at 280 nm is a result of the characteristic transition related to N-doping in CDs^33^,^47^. The broad tail is presumably originated from other functional groups on the surface of CDs^50^. The PL spectrum of the N-CD layer exhibited a dominant emission band centered at 505 nm, and the full width at half maximum was as broad as ca. 240 nm. This PL band was found to consist of five PL peaks centered at 375, 419, 490, 540, and 590 nm via multiple peak fitting process, which are denoted as A, B, C, D, and E, respectively, in Fig. 3a. The near-UV shoulder band centered at 419 nm is attributable to the transition of π electrons localized in C=C bonds and the electron transition in non-hexagonal carbon rings with *sp*^2^ structure^26^,^51^,^52^. The visible emission peak at 505 nm and shoulder at 590 nm is ascribed to the radiative recombination associated with N-related functional groups and the quantum confinement effect with diverse N-CD sizes, respectively^18^,^50^,^51^,^52^. The detailed origins of the PL emission are further described in Supplementary Information (Figure S5).

Figure 3b is the PL spectra of the QD layer, N-CD layer, and QD/N-CD bilayer, obtained at the same measurement stage. The PL data shows that the QD layer and QD/N-CD bilayer exhibited strong emission peak at 622 nm but the N-CD layer did not show an emission at 622 nm, indicating that the EL from type A QD-LEDs was emitted exclusively from QD layer (Fig. 2f). No EL blueshift was observed in PL of the QD layer, irrespective of the N-CD layer insertion. Noticeably, the PL intensity of QD/N-CD bilayer was typically several times higher than that of the QD layer, and the similar trend was also observed in the EL of QD-LEDs (Fig. 2d–f), all of which signify that the N-CD insertion layer provoked a stimulation of QD layer luminescence.

In order to examine the role of the N-CD layer for the enhanced luminescence of QD/N-CD, we compared the absorption spectrum of the QD layer and PL emission spectrum of N-CD layer (Fig. 3c). Since the spectral overlap region was clearly observed, as marked with red checker area in Fig. 3c, we envision that the energy corresponding to the luminescence of N-CD within the overlap area was possibly transferred to the QD layer in the QD/N-CD bilayer structure for the enhanced PL emission of QD, which is the FRET process^13^. For the FRET, it is essential that the transition energy value of energy donor (N-CD) is slightly higher than absorption energy of energy acceptor (QD), and the absorbance/emission spectra of QD/N-CD fulfilled such requirement (Fig. 3c). Specifically, among several PL emissions marked in Fig. 3a, the (E)-PL emission related with the quantum confinement effect of N-CD is thought to dominantly contribute to the FRET to QD layer because the (E)-PL emission and absorbance of QD have the same peak position of 590 nm wavelength. Since the absorbance energy of N-CD is higher than the luminescence energy of QD, the emitted energy from QD cannot be absorbed to N-CD in the (Figure S4). Meanwhile, if the QD luminescence enhancement was endowed by radiative energy transfer from N-CD, the PL intensity of QD would increase by only the amount absorbed from PL of N-CD, resulting in quite little PL enhancement. However, as shown in Fig. 3b, the PL intensity was significantly improved by making the QD/N-CD bilayer although the PL intensity of N-CD was quite negligible with respect to the amount of PL enhancement of the QD layer, implying the radiation-less FRET process.

According to the TEM data of the QD/N-CD interface (Figure S11, Supplementary Information), the QD layer was observed to be coated upon the N-CD layer without forming an interfacial void or air gap. Thus, it is surmised that such heterointerface of QD/N-CD was good for the FRET-assisted PL enhancement shown in Fig. 3b. Notably, since the average N-CD diameter was 4 nm, the 4 nm-thick N-CD interlayer was observed to be equivalent to mono-N-CD-thick layer with a coverage of nearly 100% on the underlayer surface.

**Figure 4:**
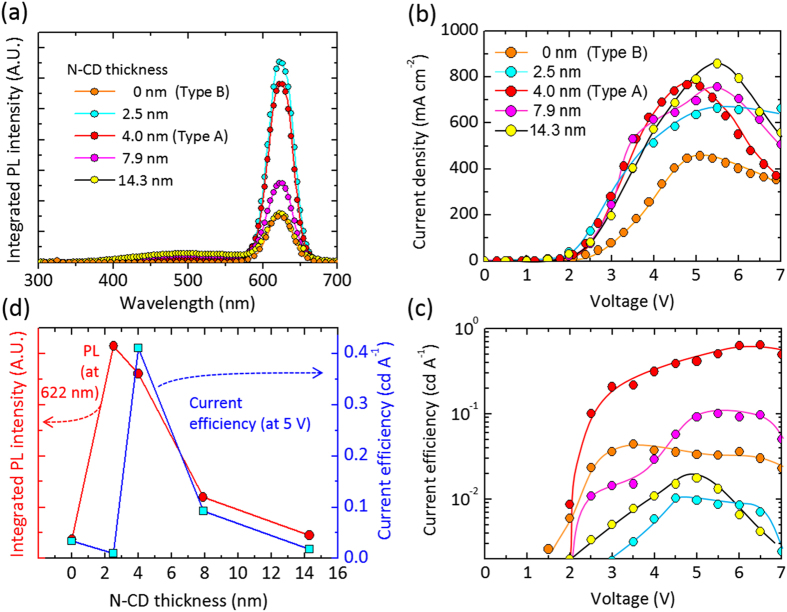
Effect of N-CD layer thickness on luminescent and electrical performances of QD-LEDs. (**a**) PL spectra of QD/N-CD bilayer with various N-CD thicknesses. (**b**) *J*–*V* and (**c**) current efficiency–voltage curves of the QD-LEDs with diverse N-CD thicknesses of 2.5–14.3 nm. (**d**) Integrated PL intensity of QD/N-CD bilayers and current efficiency of QD-LEDs plotted as a function of N-CD interlayer thickness.

Therefore, it is imperative to investigate the effect of N-CD layer thickness to find the optimal thickness for high-efficiency FRET and QD-LED performance. For this study, we varied the N-CD layer thickness from 2.5 to 14.3 nm. In Fig. 4a, the PL intensity dramatically increased when introducing the ultrathin N-CD layer with thickness of 2.5–4.0 nm, then the intensity was gently decreased as increasing the N-CD layer thickness. Finally, when the N-CD layer became quite thick (14.3 nm), the PL intensity was almost same as that of the sole QD layer, indicating that such a thick N-CD layer did not contribute to energy transfer between donor and acceptor for FRET but rather to donor–donor intra-ensemble energy transfer^14^.

We further scrutinized the effect of N-CD thickness on current density and luminous current efficiency of QD-LED structures with diverse N-CD interlayer thicknesses (Fig. 4b and c). In Fig. 4b, the *J*–*V* characteristic curves of QD-LEDs show that the electrical threshold was reduced and the current density was typically enhanced by inserting the N-CD layer, irrespective of N-CD thickness, presumably because of improved hole transport capability by the N-CD interlayer. For the current efficiency, the QD-LEDs with a 2.5 nm-thick N-CD interlayer showed poor EL performance (9.7 × 10^−3 ^cd A^−1^ at 5.0 V), tentatively because of inhomogeneous current path through the N-CD interlayer with poor coverage. As inserting 4.0 nm-thick N-CD interlayer, the QD-LEDs exhibited a luminous current efficiency of 4.1 × 10^−1^ cd A^−1^ at 5.0 V (highest current efficiency at 6.0 V in Fig. 4c: 6.3 × 10^−1^ cd A^−1^). Then, the current efficiency was decreased with further increasing the N-CD layer thickness (e.g., 9.2 × 10^−2^ and 1.7 × 10^−2^ cd A^−1^ at 5.0 V for 7.9 and 14.3 nm-thick N-CD interlayers, respectively), probably attributable to degraded FRET resulting from the donor–donor intra-ensemble energy transfer in the thick N-CD layer. This trend is in good agreement with work by Liu *et al*.^13^. In consideration of the N-CD-thickness-dependent PL and EL performances (Fig. 4d), the thickness of energy donor layer is critical for improving the luminescence efficiency of QD-LEDs, and the 4.0 nm-thick N-CD interlayer was supposed to be an optimal structure for high-efficiency QD-LEDs in this study.

### Time-Resolved Photoluminescent Characteristics of QD Layer with/without N-CD Interlayer

**Figure 5:**
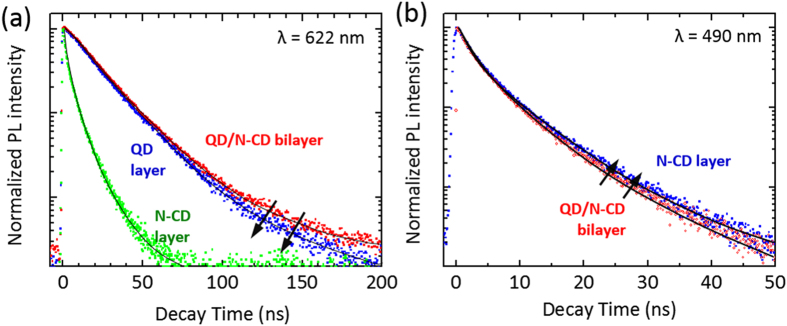
Time-resolved PL spectroscopic analysis. (**a**) TR-PL spectra of QD/N-CD, QD, and N-CD layers measured at a wavelength of 622 nm. (**b**) TR-PL spectra of N-CD and QD/N-CD layers measured at 490 nm. The decay curve of QD sole layer was negligible at wavelength of 490 nm. The corresponding fitted curves (black line) were derived from TR-PL data by an iterative deconvolution fitting process based on equation (1) and the instrumental response function. Each amplitude *W*_*i*_ and decay time *τ*_*i*_ are summarized in Table 1.

The FRET from the N-CD layer to the QD layer was further investigated by time-resolved photoluminescence (TR-PL) analyses of the N-CD layer and QD layer with/without N-CD layer. For the TR-PL analysis, the PL decays were recorded at emission wavelengths of 622 and 490 nm (Fig. 5), which are ones of the characteristic PL emission peaks of the QD and N-CD layers (shown in Fig. 5), respectively. The PL decay curves were numerically analyzed using a bi-exponential model fitting expressed by


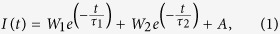


where *I, t, W*, and *τ* is normalized emission intensity, time after excitation, amplitude coefficient, and decay time constant, respectively, and the fitting results are summarized in Table 1. In Fig. 5a, the PL decay (λ = 622 nm) of the QD/N-CD bilayer dropped more slowly with respect to the QD sole layer, and the average PL lifetime of QD layer was slightly increased from 20.0 to 20.8 ns when stacking the QD/N-CD bilayer structure. This outcome suggests that the energy-acceptor QD layer was fed with the energy transferred from the energy-donor N-CD to some extent in the bilayer sample. Figure 5b shows the PL decay of the N-CD layer and QD/N-CD bilayer measured at 490 nm, exhibiting that the PL emission of the bilayer diminished faster than that of the N-CD layer because the carriers in the energy-donor N-CD layer quenched as a result of excitation energy transfer to the acceptor QD layer in the QD/N-CD bilayer. Additionally, the PL decays were recorded at an emission wavelength of 590 nm in Supplementary Information (see Supplementary Figure S12 and Table S1).

The FRET efficiency (*ξ*) was determined to be 0.13, according to the energy transfer efficiency equation of


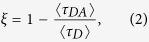


where 〈*τ*_DA_〉 and 〈*τ*_D_〉 are the amplitude-weighted average lifetime of the QD/N-CD bilayer and N-CD sole layer, respectively. Since our value (*ξ* = 0.13) is comparable to the values (*ξ* ~ 0.10–0.9) reported elsewhere, it is surmised that the enhancement of the PL intensity and increased lifetime (λ = 622 nm) of the QD/N-CD bilayer is a result of the FRET from the N-CD to the QD layer^14^,^53^,^54^.

### Electron and Hole Conduction Capability and Electronic Structure of QD-LEDs with N-CD Interlayer

**Figure 6:**
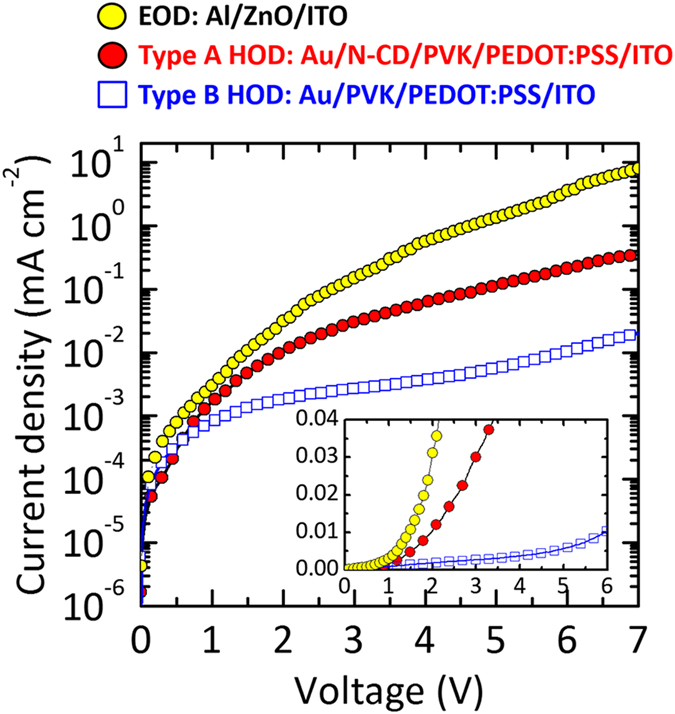
*J*–*V* characteristic curves of electron-only device (EOD) and hole-only devices (HOD) for the type A and type B QD-LEDs. The inset is the same curves plotted in a linear scale.

The high-brightness QD-LEDs with N-CD interlayer would not be only attributed to the FRET but to the high current density of the type A QD-LED structure. Hence, the electron-only device (EOD) and the HODs of Au/N-CD/PVK/PEDOT:PSS/ITO and Au/PVK/PEDOT:PSS/ITO structures with a device area of 2.0 × 2.0 mm^2^ were electrically characterized for types A and B QD-LEDs, respectively. In Fig. 6, the EOD exhibited a higher current density than those of type A and B HODs by one and two orders of magnitudes, respectively, in a typical applied voltage range of 2–7 V. This signifies that the hole conductivity plays more crucial role in determining the degree of charge carrier balance and electron overflow. The charge carrier balance and electron overflow are discussed together with the electronic structures of QD-LEDs in the later part of this sub-section. As for the HODs, the *J*–*V* curves of the HODs present very similar electrical trends with those of QD-LEDs. Type A showed a much higher current density than type B by an order of magnitude in the typical voltage range (2–7 V). This signifies that the insertion of N-CD contributed to the high hole conduction capability, yielding a higher luminance of the type A QD-LEDs (Fig. 2).

**Figure 7:**
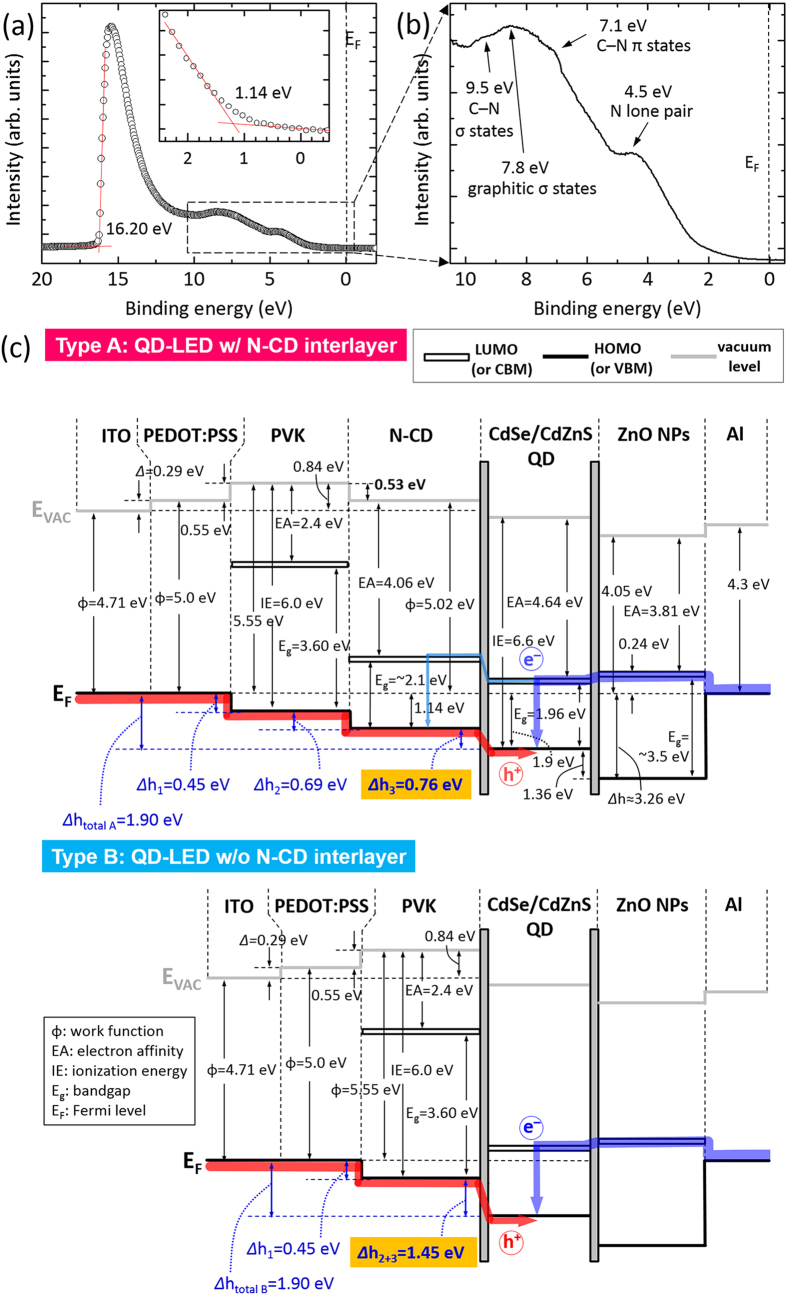
Electronic structure of QD/N-CD/PVK and QD/PVK heterojunction layers. (**a**) UPS spectrum and (**b**) valence band region UPS spectrum of N-CD. (**c**) Electronic energy level alignments of type A (w/N-CD) and type B (w/o N-CD) QD-LEDs derived from UPS spectroscopic analysis.

The high hole conduction capability of the N-CD-inserted multilayer HTL was further explored by estimating the electronic energy level alignments of the QD-LEDs, which were investigated using UPS. Work functions (*Φ*) were calculated by *Φ* = *hν* − |*E*_cutoff_ − *E*_Fermi_| (He I, *hν* = 21.22 eV). From the onset value (16.20 eV, Fig. 7a), the work function of the N-CD layer was calculated to be 5.02 eV; the valence band edge was −1.14 eV with respect to the Fermi level (inset of Fig. 7a). Thus, the ionization energy (IE) value of the N-CD layer was estimated to be 6.16 eV. The electronic structures of PVK, PEDOT:PSS, ITO, and ZnO NP layers were also characterized by the same method (Figure S8, Supplementary Information).

The UPS revealed that the N-doping significantly altered the valence band structure of CD. The UPS data in the valence band region for the N-CD layer showed a broad band including a dominant peak at 7.8 eV and three shoulder peaks at 4.5, 7.1, and 9.5 eV. As displayed in Fig. 7b, a dominant peak at 7.8 eV, related with σ bonds in graphitic structures^55^, was clearly observed. Owing to pyridinic/quaternary N formed by N-doping, two peaks at 4.5 and 7.1 eV can be ascribed to N lone-pairs and the electrons occupying the π orbitals of C–N bonds in graphene, respectively^56^; the shoulder peak at 9.5 eV is associated with the σ orbitals of C–N bonds^57^. It is noted that the peak at 3.0 eV (related to the conjugated π bonds of the C–C bonds), typically observed in undoped graphene^55^, was not clearly observed from the N-CD layer because the heavy N-doping (pyridinic N) and N-containing functional groups on the graphene surface possibly depressed the C–C 2*p*π electron states and produced the C–N 2*p*π states (~7.1 eV). The UPS (as well as XPS in Fig. 1) analysis suggests that the solution-based N-CD synthesis facilitated the heavy N-doping on the surface of graphene.

The electronic energy level alignment diagrams of QD/N-CD/PVK and QD/PVK heterojunctions were then derived from the UPS analyses for the type A (w/N-CD) and type B (w/o N-CD) QD-LEDs (Fig. 7c). The hole barrier height (*Δh*) was estimated by calculating the HOMO (or valence band maximum, VBM) positions with respect to the HOMO (or VBM) of the underlayer. The interface dipole (*Δ*) was calculated as the difference between *Φ* of the overlayer and that of the underlayer. In the control QD-LEDs (type B), the highest hole barrier was measured at 1.45 eV (=*Δh*_2+3,_ bottom panel of Fig. 7c), and noticeably the highest hole barrier could be reduced from 1.45 to 0.76 eV by inserting the N-CD layer between PVK and QD layers (=*Δh*_3,_ in type A, Fig. 7c). This implies that a more balanced electron and hole injection is expected in type A QD-LEDs, while a hole-insufficient current occurred in type B QD-LEDs. The *J*–*V* characteristics of both QD-LEDs and EOD/HODs, shown in Figs 2 and 6, respectively, support our argument. More specifically, the low electrical threshold (type A QD-LED, Fig. 2b) validates the observed electronic structure of type A QD-LEDs with a substantially reduced highest hole barrier height.

According to the *J*–*V* characteristic curves of the EOD and type A and B HODs (Fig. 6), charging and/or electron overflow may occur for both the QD-LEDs due to higher electron injection. Among the QD-LEDs, type A is expected to exhibit more balanced charge carrier injection than type B QD-LEDs due to high hole conductivity, thus less charging certainly occurs in type A QD-LEDs. As for the electron overflow, excess electrons easily overflow to N-CD layers because of low electron blocking barrier at QD/N-CD in type A QD-LEDs, then these electrons possibly contribute to the FRET process that can enhance luminescence efficiency. Of course, the charging effect would occur less in type A QD-LEDs as well. In contrast, in type B QD-LEDs, because of the rather high electron barrier built in QD/PVK and unbalanced charge carrier injection, the excess electrons cannot pass over but are seriously accumulated in the QD layer, which causes charging effect. According to our electrical and electroluminescent performance results for type A QD-LEDs, it is plausible that i) more balanced charge carrier injection and ii) less charging effect played more crucial role than the carrier confinement (in QD active layer) for high light efficiency of type A QD-LEDs. Furthermore, since such non-confined electrons (or overflowed electrons) contributed to the FRET process, type A QD-LEDs possibly showed much greater enhanced electroluminescence efficiency. Thus, we surmise that the QD/N-CD layer capable of resonance energy transfer would be a key to high-efficiency QD-LEDs.

### How the FRET Occurs in the QD-LEDs with N-CD Interlayer

**Figure 8:**
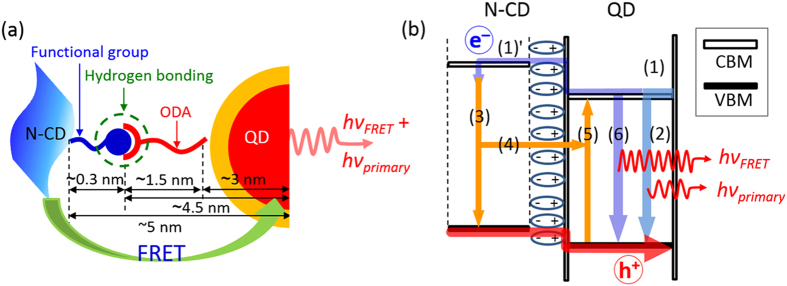
FRET from N-CD to QD. (**a**) Schematic illustration depicting the interfacial structure of QD/N-CD where the FRET occurred. (**b**) The FRET-enhanced EL process in the electronic structure of QD/N-CD heterojunction. (1) charge transport; (2) primary EL emission by exciton recombination in QDs; (1)′ electron overflow to N-CD layer; (3) non-radiative recombination; (4) FRET; (5) FRET-driven excitation and (6) EL emission.

It is well known that two major necessary conditions should be fulfilled for the FRET process: (i) the spectral overlap between donor emission and acceptor absorption peaks (already shown in Fig. 3c), and (ii) small donor-to-acceptor distance within Förster radius of ~1–10 nm that enables the interface dipole–dipole interaction. As schematically depicted in Fig. 8a, the distance between QD and N-CD layer is ca. 5 nm where the complementary dipole–dipole interaction arises from hydrogen bonding between the octadecylamine (ODA)-ligand of QD and the nitrogen (or oxygen)-related functional groups of N-CD^58^ (Figs S6 and S7, Supplementary Information). The Förster distance (*R*_0_), at which FRET efficiency (*ξ*) is 0.5, is calculated at 5.4 nm for our system, thus the QD-to-N-CD distance is in good accordance with the *R*_0_ for the efficient FRET^59^. In addition, the FRET rate (*k*_FRET_) was calculated to be 2.9 × 10^7^ s^−1^, comparable to values reported elsewhere^13^. Hence, we surmise that the spectral overlap of the N-CD and QD layers and the short range QD-to-N-CD distance are responsible for the observed FRET.

One more basic condition for the FRET is the electron–hole recombination at the energy donor N-CD layer, as schematically depicted in electronic structure of Fig. 8b. The electrons and holes injected to the QD layer recombines for EL emission, referred to as (1) and (2) process in Fig. 8b. For the FRET-driven recombination, the electrons should pass over the QD layer and be sufficiently supplied to the N-CD layer [(1)′]. According to the electronic structure derived from UPS analysis (Fig. 7c), the energy level offset of conduction band minimum (CBM) at QD/N-CD heterointerface is as small as 0.9 eV (upper panel of Fig. 7c). Due to such low electron blocking barrier height, the excess electrons possibly overflow across the small band offset of 0.9 eV into the N-CD layer [(1)′]. The overflowing electrons easily facilitate (3) either radiative or non-radiative recombination; (4) radiation-less recombination provokes the FRET from N-CD to QD. Then, (5) the FRET-driven excitation induces (6) more radiative recombination (or EL) at the QD layer, causing luminescence (or EL) enhancement.

## Conclusion

This study investigated the roles of N-CD interlayer inserted between the HTL and QD layers for high-efficiency QD-LEDs. For the electrical properties, the N-CD interlayer was found to increase the hole conduction capability and lower the highest hole barrier height, which were validated via the HOD measurement and UPS analysis, respectively. Specifically, the current density was increased by at least an order of magnitude for the N-CD inserted HODs and QD-LEDs. Moreover, the N-CD interlayer acting as a FRET energy-donor layer to the QD layer, made possible by the spectral overlap and small band offset, enhanced the luminescence of the QD layer. The increased PL lifetime of QD and decreased PL lifetime of N-CD validated the FRET. The electronic structure of N-CD-inserted QD-LED, plotted from UPS analysis, also represented that the small difference of bandgap and band offset of QD/N-CD facilitated the FRET. Despite the all-solution-processed method, significant improvement of QD-LED performance was demonstrated when utilizing the N-CD interlayer. Hence, we believe that the ability of the N-CD interlayer which can simultaneously enhance the electrical and luminescent performances of QD-LEDs would be readily exploited for many other QD-based high-efficiency optoelectronic devices.

## Methods

### Syntheses of N-doped carbon dot and ZnO nanoparticle

N-CD was synthesized by the carbonization process in ethanolamine (3 mL) and hydrogen peroxide (H_2_O_2_, 4.5 mL) aqueous solution of 2:3 volume ratio at 200 °C for 120 min^60^. During the synthesis procedure, the color of ethanolamine changed into bright yellow just after adding H_2_O_2_ and became dark (black gel without sediment) at last. Then, the dark colloidal N-CDs were dispersed in deionized water:2-methoxyethanol (1:80) mixture solvent. This dispersed solution was used for spin-coating of the N-CD layer.

ZnO NPs were synthesized using precursors of zinc acetate dihydrate and tetramethylammonium hydroxide (TMAH) via a sol–gel process. TMAH (0.55 M), dissolved in ethanol, was added dropwise to 0.1 M zinc acetate dihydrate dissolved in dimethyl sulphoxide (DMSO), followed by stirring for 1 h in an ambient air. The ZnO NP was then rinsed and dispersed in ethanol, at a concentration of ~30 mg mL^−1^. The mean size of the ZnO NP was measured at ~5 nm.

### QD-LED fabrication

QD-LEDs were fabricated using a solution spin-coating process on 180-nm-thick indium-tin oxide (ITO)-coated glass substrates (sheet resistance: 10 Ω/sq). The light-emitting area was 2.0 × 2.0 mm^2^, defined by the area of patterned ITO and aluminum (Al) electrodes. For type A and B QD-LEDs, HTL heterojunctions of N-CD/PVK/PEDOT:PSS and PVK/PEDOT:PSS were deposited by the spin-coating method on the patterned ITO/glass substrates, as schematically depicted in Fig. 2a, respectively. The PEDOT:PSS (Heraeus, Clevios™ P VP.AI 4083), PVK, and N-CD layers were consecutively spin-coated on the substrate at spin speed of 3000, 3000, 2000 rpm, and were cured at 190, 180, and 150 °C for 30 min, 1hr, and 30 min in an air ambient, respectively, after each of the spin-coating processes. Specifically, the PVK (Aldrich, average molecular weight: ~120,000), dissolved in chlorobenzene (12.5 mg mL^−1^), was filtered by a polyvinylidene difluoride filter (mean pore size: 0.45 μm) before the spin-coating process. The CdSe/CdZnS core/shell QDs with octadecylamine ligand (QD Solution, Nanodot-HE100), dispersed in toluene with a concentration of 25 mg 10 mL^−1^, were spin-coated on the HTL heterojunctions at 1000 rpm for 30 s, followed by dry-out. The mean size of QD is ca. 6 nm, and the QY of the QDs is ca. 85%. The ZnO NP, dispersed in ethanol, was then spin coated onto the QD layer, and annealed at 125 °C. For the cathode layer, Al was deposited through a shadow mask using thermal evaporation technique. It is noted that all the QD-LEDs were processed using the spin-coating method under ambient air conditions (20–24 °C and 10–30% humidity condition), except for the electrode depositions, and were encapsulated with a glass lid using an UV curable epoxy resin.

### Characterizations

The structural properties and surface morphologies of the N-CD layer were characterized by micro-Raman spectroscopy (Renishaw, excitation wavelength of 514 nm) and field emission scanning electron microscopy, respectively. A bright-field HR-TEM, with an acceleration voltage of 200 keV (JEOL, JEM-2100F), was used to inspect the mean diameter and crystallinity of N-CDs as well as to determine the thickness of each layer. To confirm the formation of N-CD, Fourier transform infrared spectroscopy (FT-IR; Perkin Elmer, Spectrum One) analysis was performed, and photoelectron spectroscopic analysis was carried out in an ultrahigh vacuum (<5 × 10^−10^ Torr) using monochromatic Al Kα X-ray (hν = 1486.8 eV) for XPS. Optical absorbance was characterized using an UV–visible–near-infrared spectrometer (Agilent Technologies, Cary 5000). For time-integrated PL characterizations, a He–Cd laser (*λ* = 325 nm & 15 mW, IK5551R-F, Kimmon Koha Co., Ltd.) was used as the excitation source. Electrical and electroluminescent properties of the QD-LEDs were characterized by *J*–*V*–*L* measurements and EL spectroscopy (Spectra Scan PR-670 spectroradiometer & Keithley, 2601 sourcemeter). The hole conduction capability of the QD-LEDs were further characterized by measuring the electrical properties of HODs with/without N-CD interlayer. The TR-PL of QD, N-CD, and QD/N-CD were measured using a time correlated single photon counting system (Horiba Jobin Yvon iHR320). A pulsed InGaN multiple quantum-well LED emission (λ = 405 nm, repetition rate 1 MHz and optical pulse duration 200 ps) was employed as an excitation source for the TR-PL measurement. The work function of each layer and the energy level alignment of the QD-LEDs were determined by measuring the secondary electron cutoff and the HOMO regions of the UPS spectra, respectively. The UPS spectra were recorded with He I radiation (*hv* = 21.22 eV), and to obtain the low-energy secondary electron cut-off, a bias voltage of −10 V was applied to the sample under normal emission geometry.

## Additional Information

**How to cite this article:** Park, Y. R. *et al*. Quantum-Dot Light-Emitting Diodes with Nitrogen-Doped Carbon Nanodot Hole Transport and Electronic Energy Transfer Layer. *Sci. Rep.* **7**, 46422; doi: 10.1038/srep46422 (2017).

**Publisher's note:** Springer Nature remains neutral with regard to jurisdictional claims in published maps and institutional affiliations.

## Supplementary Material

Supplementary Information

## Figures and Tables

**Table 1:** Photoluminescence decay parameters of the QD/N-CD, QD, and N-CD layers derived from the equation (1).

Emission wavelength (nm)	490		622	
Layer	N-CD	QD/N-CD	QD	QD/N-CD
A	5.0	13.7	12.5	8.9
W__1__(%)	70.0	75.0	53.8	50
τ__1__ (ns)	2.8	2.4	14.3	14.6
W__2__(%)	30.0	25.0	46.2	50.0
τ__2__ (ns)	11.1	11.1	26.7	27.0
**<τ>** (**ns**)	**5**.**3**	**4**.**6**	**20**.**0**	**20**.**8**

The emission wavelength was adjusted to the donor and acceptor emission, respectively. The sum of the individual amplitude *W*_*i*_ is normalized to unity and <*τ*> =∑ *w*_*i*_ ∙ *τ*_*i*_ is the amplitude-weighted average decay time.
