# Supplementary material for: Quantum-Dot Light-Emitting Diodes with Nitrogen-Doped Carbon Nanodot Hole Transport and Electronic Energy Transfer Layer
Source: Sci Rep. 2017 Apr 12;7:46422. doi: 10.1038/srep46422 (PMC5388879; doi:10.1038/srep46422)
Supplement: Supplementary Information [file srep46422-s1.pdf]

Supplementary Information for

Quantum-Dot Light-Emitting Diodes with Nitrogen-Doped Carbon Nanodot Hole Transport and Electronic Energy Transfer Layer

Young Ran Park[1], Hu Young Jeong[2], Young Soo Seo[3], Won Kook Choi[4], & Young Joon Hong[1,3,*]

[1]Graphene Research Institute, Sejong University, Gwangjin-gu, Seoul 05006, Republic of Korea.

[2]UNIST Central Research Facilities (UCRF), UNIST, Ulsan 44919, Republic of Korea.

[3]Department of Nanoscience and Advanced Materials Engineering, Sejong University, Gwangjin-gu, Seoul 05006, Republic of Korea.

[4]Materials and Life Science Research Division, KIST, Seongbuk-gu, Seoul 02792, Republic of Korea.

*Correspondence and requests for materials should be addressed to Y.J.H. (E-mail: yjhong@sejong.ac.kr)

This file includes:

Supplementary Figures 1–13;

Supplementary Table 1;

Supplementary Descriptions;

Supplementary References

S1. SEM inspection of multilayer HTLs

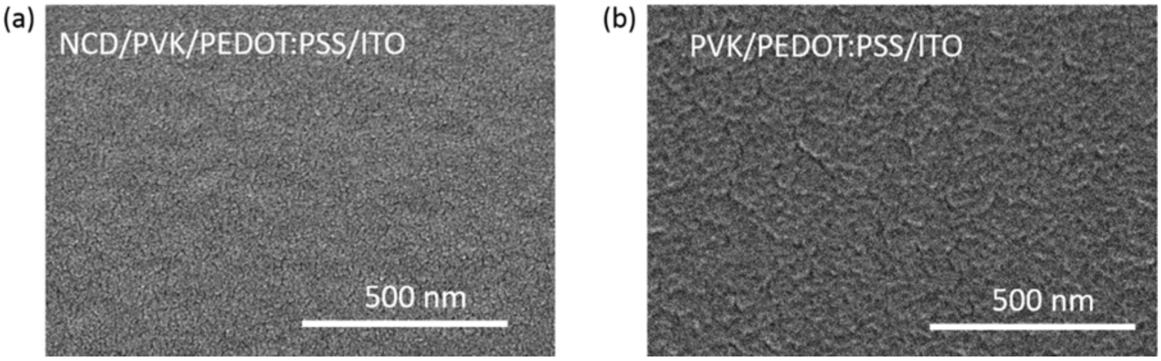

Figure S1. 45°-tilt-view SEM images of (a) N-CD/PVK/PEDOT:PSS/ITO and (b) PVK/PEDOT:PSS/ITO layers.

Figures S1a and 1b are SEM images displaying the surface morphologies of N-CD-coated PVK/PEDOT:PSS/ITO and PVK/PEDOT:PSS/ITO. It is noted that no significant degradation or damage was observed after coating of each layer, as confirmed by UPS analysis. The SEM inspection revealed that the N-CD-coated layer (Figure S1a) exhibited a much smoother surface than the PVK/PEDOT:PSS/ITO layers (Figure S1b), which can be an important factor for allowing uniform hole conducting paths responsible for high current capacity of type A QD-LED.

S2. Molecular structure of N-atom-doped CDs

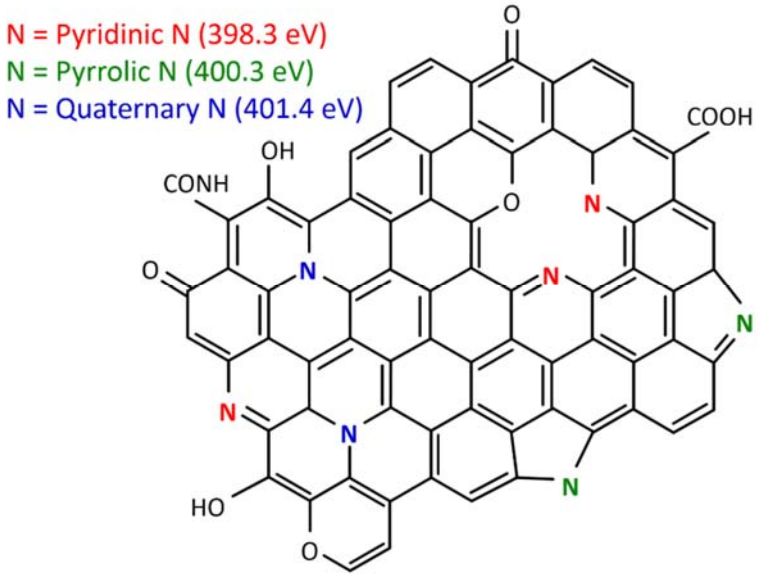

Figure S2. Schematic representation of three configurations of atomically doped N (pyridinic, pyrrolic, and quaternary N) in graphene[1].

The N atoms can be doped into the basal (or surface) plane of a graphene sheet in several different forms. The schematic illustration of Figure S2 shows atomic configurations of doped N in graphene (or CD layer), which are pyridinic, pyrrolic, and quaternary N. The characteristic XPS peaks for pyridinic, pyrrolic, and quaternary N are located at about 398.3, 400.3, and 401.4 eV, respectively, as shown in Figure 1e. It is known that the electron density in pyridinic and pyrrolic N-doped graphene is much less than undoped graphene[2]. Additionally, the N-related functional group acts as a *p*-type acceptor impurity. Especially, in Figure 1f, the integrated peak intensities of pyridinic, pyrrolic, and functional group N are higher than that of quaternary N, thus our N-CD with electron deficiency is thought to behave *p*-type conductor, similar to the findings in N-doped carbon nanotubes[3].

S3. C 1*s* and O 1*s* XPS results of the N-atom-doped CD layer

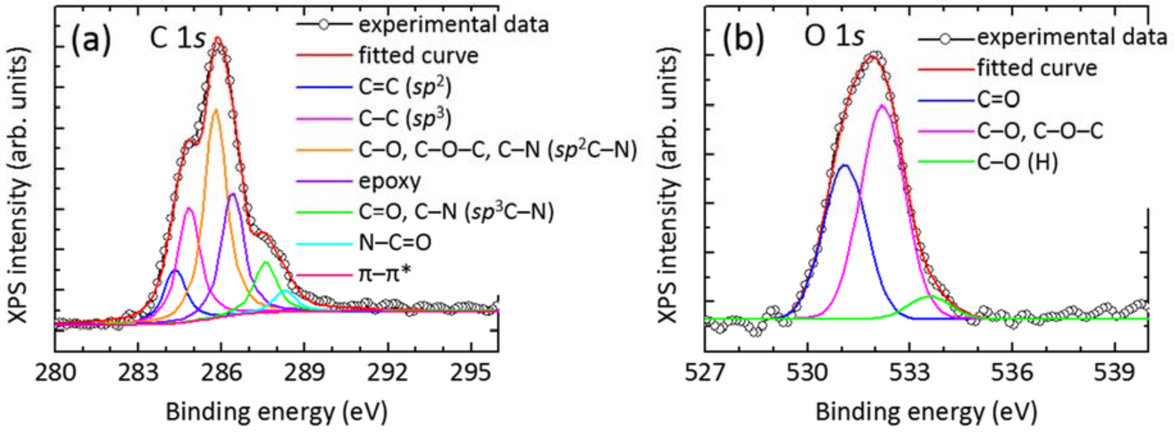

Figure S3. Peak-fitted (a) C 1*s* and (b) O 1*s* XPS spectra of N-CD layer. The spectra were de-convoluted using Doniach–Šunjić functions with Lorentzian line shapes.

Figure S3a shows the C 1*s* spectrum of the N-CD exhibiting strong multiple peaks at above 285 eV, which is noticeably distinguished from nearly symmetric C 1*s* peak of high-quality undoped graphene[4,5]. The C 1*s* band was de-convoluted into the following characteristic peaks: carbon in graphite (sp^2 C=C, 284.3 eV; sp^3 C–C, 284.8 eV); carbon singly bound to oxygen in phenols and ethers (i.e., C–O, C–O–C, C–N(sp^2C–N), 285.6 eV); epoxy bound to graphene (i.e., oxygen atom joined by single bonds to two adjacent carbon atoms), thus forming the three-membered epoxide ring (286.8 eV); carbon doubly bound to oxygen in ketones, quinones, and esters (i.e., C=O, C=N, 287.6 eV and –O–C=O, –N–C=O(sp^3C–N), 288.4 eV); the characteristic shake-up line of carbon in aromatic compounds (π–π* transition, 291.2 eV)[1,6]. The peak-fitted C 1*s* spectrum shows that the fraction of C–N (sp^2C–N, sp^3C–N) as well as that of sp^3 C–C, C–O, C=O, and O–C=O increased, while the sp^2 C=C fraction decreased in accordance with O- and/or N-doping (and functionalization). In consideration of amide (–N–C=O–) peak, the CONH groups were formed on the N-CD to some extent using our N-CD synthesis method. The FT-IR data (Figure 1d in main text) also support the argument.

Figure S3b shows that the deconvolution of the O 1*s* spectrum resulted in three peaks: oxygen doubly bound to carbon (i.e., O=C, 531.1 eV); oxygen singly bound to carbon (i.e., O–C, C–O–C, 532.1 eV); oxygen in hydroxyl group (C–O(H), 533.6 eV). Since the peaks of O–N or O=N functional groups

were not clearly observed in N 1*s* peak analysis (Figure 1f), it is plausible that the two peaks (at 531.1 and 532.1 eV) are responsible for O atoms in esters, carboxyls, anhydrides, and pyrones with O–C and/or O=C.

S4. Optical properties of N-CD and QD

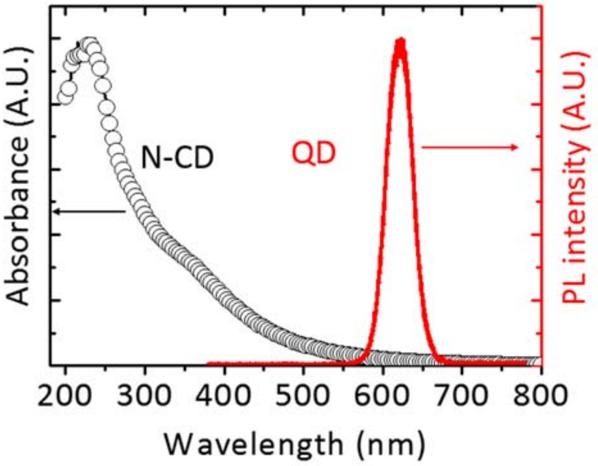

Figure S4. UV–Vis absorption spectrum of the N-CD layer and PL spectrum of the QD layer.

Figure S4 shows the UV–Vis absorption spectrum of the N-CD layer and the PL spectrum of the QD layer. The QD yielded a sharp recombination spectrum with a peak centered at 622 nm, while the spectrum of N-CD contained a broad absorption tail originated from N- or O-related functional groups at the surface of N-CD. Since the spectral overlap region was not observed in Figure S4, the luminescence of QD was not surmised to be absorbed to N-CD in the QD/N-CD bilayer.

S5. Peak-fitted PL spectrum of the N-atom-doped CDs

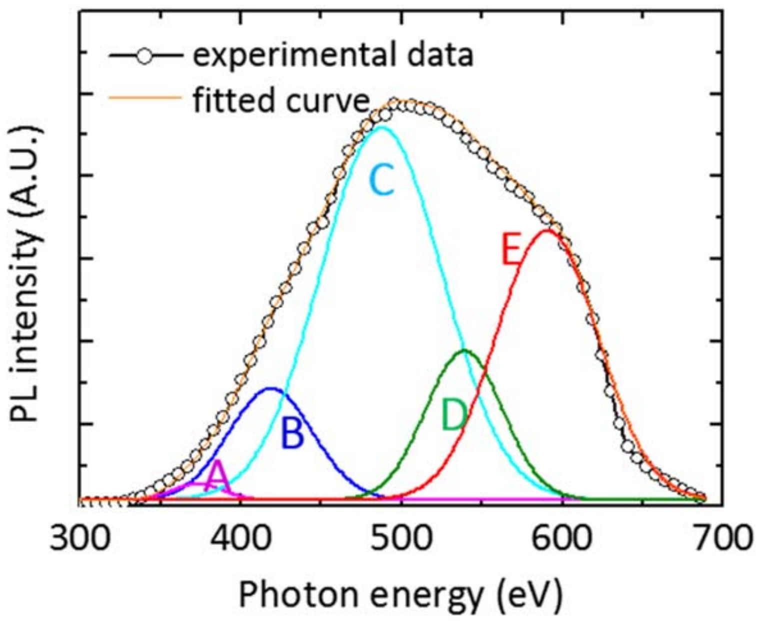

Figure S5. Peak-fitted photoluminescence spectrum of the N-CD layer. The spectra were de-convoluted with Gaussian line shapes.

To date, the PL emission of N-CD has been intensively studied, which referred the quantum size effect[7-9], zigzag sites[10], different degree of π–conjugation[11,12], edge states[13], surface defects[14,15], surface groups[16], N-doping[1,9,17], and surface passivation[18]. Figure S5 shows that PL band for the N-CD layer, including five Gaussian-fitted peaks at 375 nm (UV), 419 nm (blue), 490 nm (cyan), 540 nm (green), and 590 nm (orange), denoted as A, B, C, D, and E, respectively. The weak UV emission peak (A) is assigned to the transition of π electrons localized in C=C bonds; the blue emission (B) is from the electron transition of the surface-related topological defects (non-hexagonal rings such as pentagonal, heptagonal or octagonal rings) within the sp^2 clusters, in consistence with the previous reports[17,19-21]; the PL color distribution from cyan (C) to orange (E) is presumably resulted from the intrinsic state/interstate to band transitions associated N-related (primary amines and amide) functional group and the N-dopant[1,9,12,16,17,22-24] and/or quantum confinement effect of CDs [9,16,20,22-26].

S6. Interfacial chemical structure of QD/N-CD for the FRET

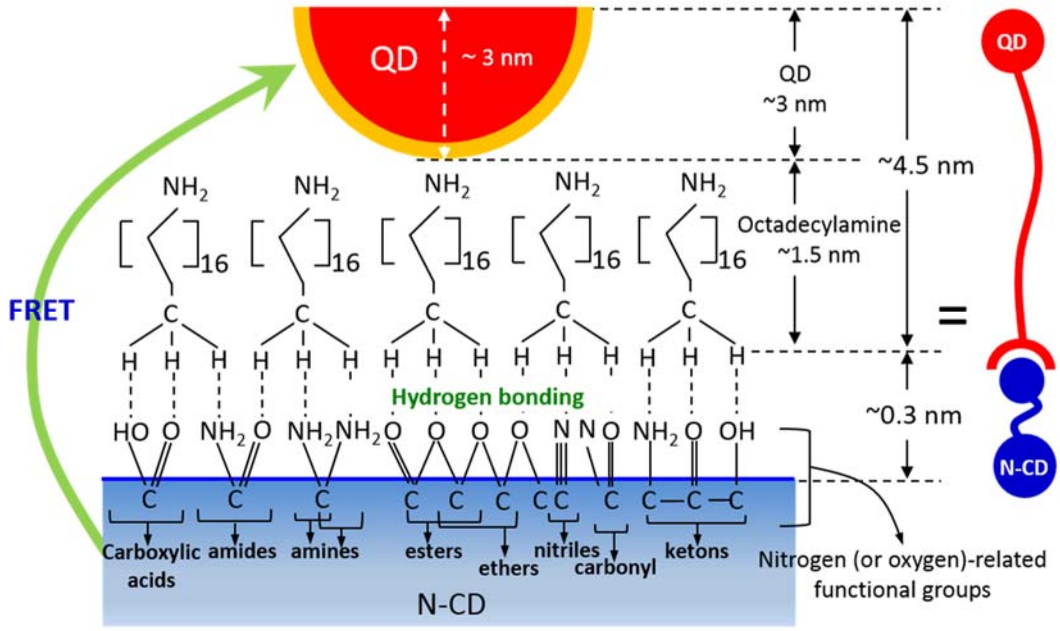

Figure S6. Schematic illustration of heterointerfacial checmical structure of QD/N-CD for the FRET. The functional groups on N-CD and surface octadecylamine-ligands (tilted length~1.5 nm)[27] of QD are mutually linked by hydrogen bonds within a short range distance.

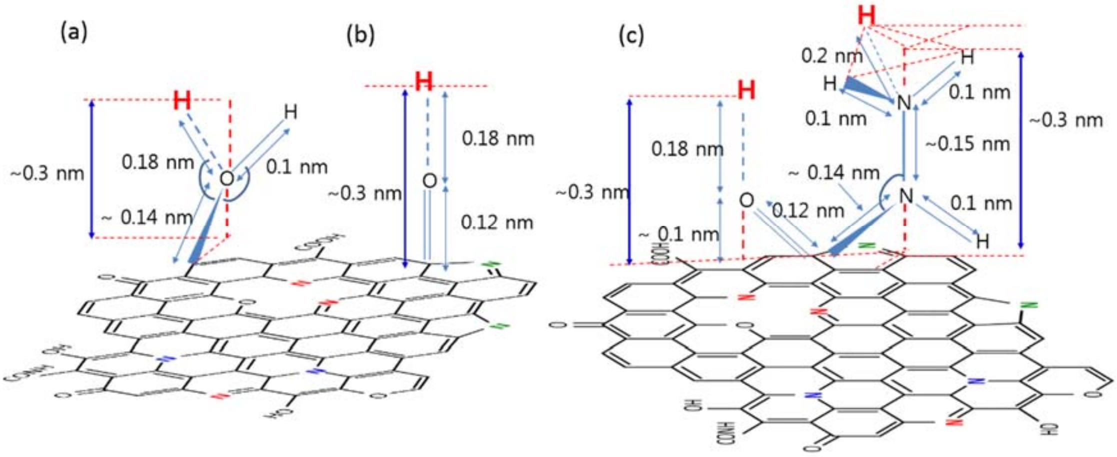

Figure S7. Illustration for covalent and non-covalent hydrogen bond lengths between functional group of N-CD and ligands of QD. (a) C–OH, (b) C=O, and (c) C–NHNH$_2$ are represented as typical functional groups for N-CDs. Cyan dotted line denotes non-covalent hydrogen bond; red-dotted line is drawn for determining the normal distance to the graphene surface. The bold, red H signifies hydrogen atom in the ligand tail of QD. The distance from QD ligand to CD is estimated to be ~0.3 nm.

S7. UPS analyses of multilayer HTLs

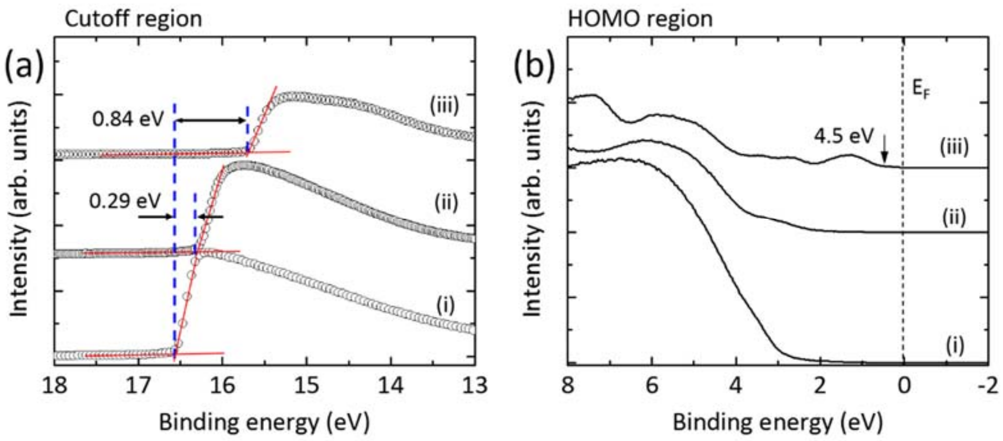

Figure S8. UPS spectra obtained in the (a) low kinetic energy region (the secondary electron cutoff) and (b) the low-binding-energy region (the highest occupied molecular orbital (HOMO) region) for (i) ITO, (ii) PEDOT:PSS/ITO, (iii) PVK/PEDOT:PSS/ITO.

To examine the electronic structure and electronic energy level alignments of the QD-LEDs, a UPS measurement was carried out in the secondary electron cutoff and HOMO regions for the PVK/PEDOT:PSS/ITO heterostructures. As shown in Figure S8a, the cutoff of secondary electrons was determined by the intersection of two tangential lines extended from the background and downward cutoff curves. The onset values of the secondary electrons from the (ii) PEDOT:PSS and (iii) PVK were lower with respect to those of (i) ITO and (ii) PEDOT:PSS by 0.29 and 0.55 eV; the differences correspond to the interface dipole barrier (*Δ*) at PEDOT:PSS/ITO and PVK/PEDOT:PSS heterointerfaces, respectively (Figure 7c and Figure S8a). From the onset values, the work function of each layer were calculated to be 4.71, 5.00, and 5.55 eV for (i) ITO, (ii) PEDOT:PSS/ITO, and (iii) PVK/PEDOT:PSS/ITO layers, respectively. The HOMO edges of PVK was 0.45 eV with respect to the Fermi level (Figure S8b), and thus, the ionization energy of PVK was estimated to be the value of 6.0 eV (= 0.45 + 5.55).

S8. Cross-sectional TEM & EDS analysis of N-CD inserted QD-LEDs

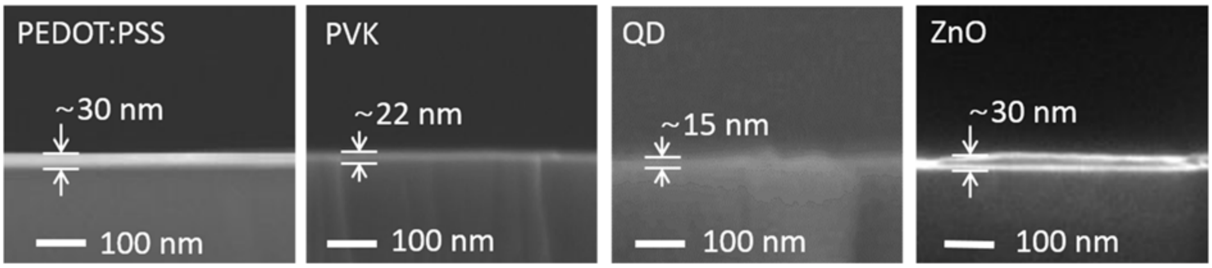

Figure S9. Cross-sectional SEM images of PEDOT:PSS, PVK, QD, and ZnO layers (from left to right) coated on ITO/glass substrate.

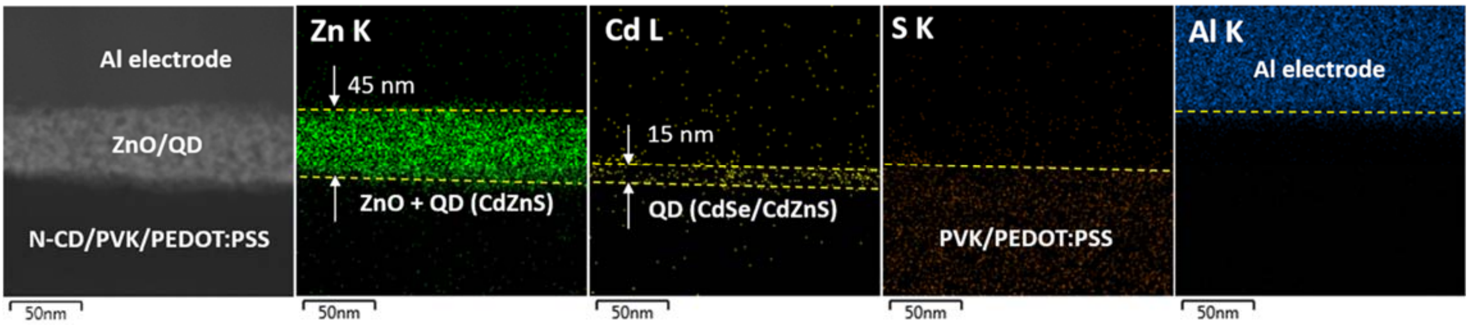

Figure S10. Cross-sectional TEM (leftmost) and the corresponding EDX mapping images for elements of Zn, Cd, S, and Al (from left to right).

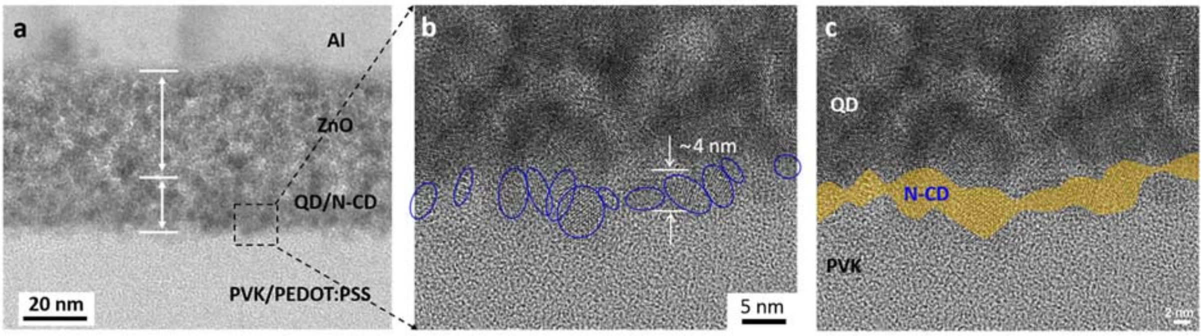

Figure S11. (a) Low magnification TEM image of the cross-sectional QD-LED structure. (b) High resolution image magnified from the boxed area marked in (a). (c) The corresponding false-colored illustration for N-CD layer, drawn according to TEM analysis of (b) image.

In order to measure the thickness of each layer, we complimentarily employed scanning electron microscopy and transmission electron microscopy. For the electron microscopic inspections, the samples were cross-sectioned by ion milling machine. Roughly, the thickness of each layer was

measured: the thickness of PEDOT:PSS, PVK, QD, and ZnO was ca. 30, 22, 15, and 30 nm, respectively (Figure S9). From combined TEM and EDX measurements (Figure S10), the thickness of ZnO/QD was measured at 45 nm by mapping Zn element, and the thickness of Cd-content layer (QD layer) was 15 nm. Thus, the ZnO layer is estimated to be 30 nm. From high-magnification TEM inspection, the average thickness of N-CD layer was estimated to be 4–5 nm, as shown in Figures S11b and 11c.

S9. Time-Resolved Photoluminescent Characteristics of QD/N-CD Layer

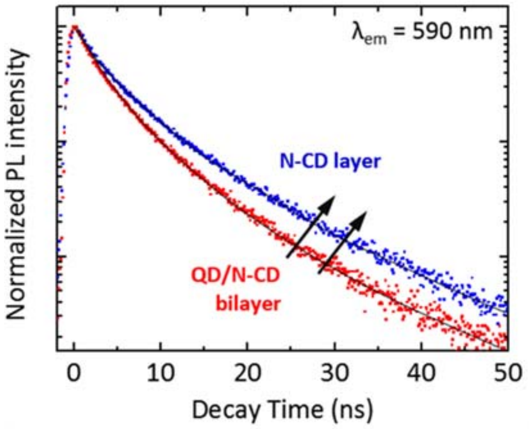

Figure S12. Time-resolved PL spectroscopic analysis. TR-PL spectra of N-CD and QD/N-CD layers measured at 590 nm.

For the TR-PL analysis, the PL decays were recorded at emission wavelengths of 590 nm (Figure S12), which are one of the characteristic PL emission peaks of the N-CD layers (shown in Figure 3a) in the spectral overlapping region between QD absorption and N-CD PL emission. The PL decay curves were numerically analyzed using a bi-exponential model fitting and the fitting results are summarized in Table S1. Figure S12 shows the PL decay of the N-CD layer and QD/N-CD bilayer measured at 590 nm, exhibiting that the PL emission of the bilayer diminished faster than that of the N-CD layer because the carriers in the energy-donor N-CD layer quenched as a result of excitation energy transfer to the acceptor QD layer in the QD/N-CD bilayer. The FRET efficiency (ξ) was determined to be 0.19 and the FRET rate (k_{FRET}) was calculated to be 4.5 $\times 10^7$ s^{-1}.

S10. Effect of annealing temperature on electrical and electroluminescent performances of QD-LEDs

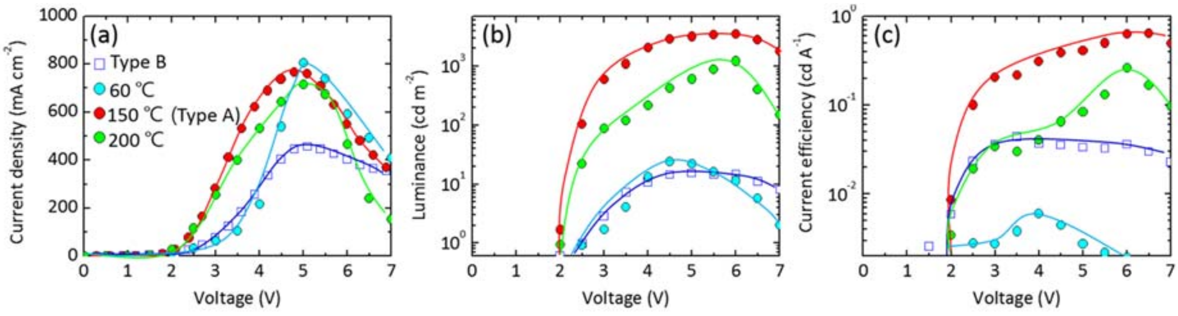

Figure S13. Electrical and EL performances of the QD-LEDs treated at diverse annealing temperature. (a) *J*–*V* characteristic curves of the QD-LEDs. Plots of (b) luminance and (c) current efficiency as a function of applied bias voltage.

It is imperative to optimize the annealing temperature for high-efficiency QD-LEDs because too high temperature degrades (or oxidizes) as-coated layers while too low temperature does not sufficiently de-gas the organic solvent and residual moisture. It is well known that 2-methoxyethanol used as solvent of N-CD is evaporated over 125 °C. If the annealing is carried out above 250 °C, PSS in PEDOT:PSS can be certainly degraded, as reported elsewhere[28,29]. Also, PVK is known to be highly degraded over 300 °C[30]. Considering the literatures, the annealing temperature of 125–250 °C may be desirable.

For optimizing the annealing temperature, we carried out the annealing at 60, 150, and 200 °C after spin coating of each layer, and compared the device performances. In Figure S13, the QD-LEDs annealed at 150 °C showed the highest current density, luminance, and current efficiency, representing superior QD-LED performances. We believe that 60 °C was insufficient to vaporize the solvent and moisture that can degrade device performances. The annealing temperature of 200 °C was found to be better than 60 °C, but was inferior to 150 °C in terms of luminance and current efficiency. Since we performed all the spin-coating and annealing procedures under normal air ambient condition, too high temperature (200 °C) might possibly oxidize the surface of as-coated layer. Hence, we believe that the annealing at 150 °C was most effectual for removing the moisture and organic solvent with little degradation among a series of annealing experiments shown in Figure S13.

Table S1. Photoluminescence decay parameters of the N-CD and QD/N-CD layers derived from the equation (1) in the main text. The emission wavelength was adjusted to the donor and acceptor emission, respectively. The sum of the individual amplitude W_i is normalized to unity and $\langle\tau\rangle = \sum w_i \cdot \tau_i$ is the amplitude-weighted average decay time.

Emission wavelength (nm)	590	
Layer	N-CD	QD/N-CD
A	6.2	2.7
W_1 (%)	75.4	90
τ_1 (ns)	3.4	3.1
W_2 (%)	24.6	10
τ_2 (ns)	11.1	11.1
**$\langle\tau\rangle$ (ns)**	**5.3**	**4.3**

Supplementary References

1. Tang, L.B., Ji, R.B., Li, X.M., Teng, K.S. & Lau, S.P. Energy-level structure of nitrogen-doped graphene quantum dots. *J. Mater. Chem. C* **1**, 4908–4915 (2013).
2. Wang, C.D. et al. In situ nitrogen-doped graphene grown from polydimethylsiloxane by plasma enhanced chemical vapor deposition. *Nanoscale* **5**, 600–605 (2013).
3. Lim, S.H., Li, R.J., Ji, W. & Lin, J.Y. Effects of nitrogenation on single-walled carbon nanotubes within density functional theory. *Phys. Rev. B* **76**, 16 (2007).
4. Kumar, A., Voevoclin, A.A., Zemlyanov, D., Zakharov, D.N. & Fisher, T.S. Rapid synthesis of few-layer graphene over Cu foil. *Carbon* **50**, 1546–1553 (2012).
5. Zhang, W.H., Nefedov, A., Naboka, M., Cao, L. & Woll, C. Molecular orientation of terephthalic acid assembly on epitaxial graphene: NEXAFS and XPS study. *Phys. Chem. Chem. Phys.* **14**, 10125–10131 (2012).
6. Favaro, M. et al. TiO2/graphene nanocomposites from the direct reduction of graphene oxide by metal evaporation. *Carbon* **68**, 319–329 (2014).
7. Sun, Y.P. et al. Quantum-sized carbon dots for bright and colorful photoluminescence. *J. Am. Chem. Soc.* **128**, 7756–7757 (2006).
8. Kwon, W. et al. Electroluminescence from Graphene Quantum Dots Prepared by Amidative Cutting of Tattered Graphite. *Nano Lett.* **14**, 1306–1311 (2014).
9. Sk, M.A., Ananthanarayanan, A., Huang, L., Lim, K.H. & Chen, P. Revealing the tunable photoluminescence properties of graphene quantum dots. *J. Mater. Chem. C* **2**, 6954–6960 (2014).
10. Zheng, H.Z. et al. Enhancing the luminescence of carbon dots with a reduction pathway. *Chem. Commun.* **47**, 10650–10652 (2011).
11. Bourlinos, A.B. et al. Luminescent Surface Quaternized Carbon Dots. *Chem. Mater.* **24**, 6–8 (2012).
12. Gu, J. et al. High-yield synthesis of graphene quantum dots with strong green photoluminescence. *RSC Adv.* **4**, 50141–50144 (2014).
13. Lingam, K., Podila, R., Qian, H.J., Serkiz, S. & Rao, A.M. Evidence for Edge-State Photoluminescence in Graphene Quantum Dots. *Adv. Funct. Mater.* **23**, 5062–5065 (2013).
14. Hu, S.-L. et al. One-step synthesis of fluorescent carbon nanoparticles by laser irradiation. *J. Mater. Chem.* **19**, 484–488 (2009).
15. Xu, Q.F. et al. Single-Particle Spectroscopic Measurements of Fluorescent Graphene Quantum Dots. *Acs Nano* **7**, 10654–10661 (2013).
16. Tetsuka, H., Nagoya, A., Fukusumi, T. & Matsui, T. Molecularly Designed, Nitrogen-Functionalized Graphene Quantum Dots for Optoelectronic Devices. *Adv. Mater.* **28**, 4632–4638 (2016).
17. Tang, L.B. et al. Deep Ultraviolet to Near-Infrared Emission and Photoresponse in Layered N-Doped Graphene Quantum Dots. *ACS Nano* **8**, 6312–6320 (2014).
18. Baker, S.N. & Baker, G.A. Luminescent Carbon Nanodots: Emergent Nanolights. *Angew. Chem.* **49**, 6726–6744 (2010).
19. Tang, L.B. et al. Deep Ultraviolet Photoluminescence of Water-Soluble Self-Passivated Graphene Quantum Dots. *ACS Nano* **6**, 5102–5110 (2012).
20. Gan, Z.X. et al. Mechanism of Photoluminescence from Chemically Derived Graphene Oxide: Role of Chemical Reduction. *Adv. Opt. Mater.* **1**, 926–932 (2013).
21. Yang, P. et al. Facile synthesis and photoluminescence mechanism of graphene quantum dots. *J. Appl. Phys.* **116**, 244306 (2014).

22. Fang, Y. et al. Easy Synthesis and Imaging Applications of Cross-Linked Green Fluorescent Hollow Carbon Nanoparticles. *Acs Nano* **6**, 400–409 (2012).
23. Jin, S.H., Kim, D.H., Jun, G.H., Hong, S.H. & Jeon, S. Tuning the Photoluminescence of Graphene Quantum Dots through the Charge Transfer Effect of Functional Groups. *ACS Nano* **7**, 1239–1245 (2013).
24. Tetsuka, H., Nagoya, A. & Asahi, R. Highly luminescent flexible amino-functionalized graphene quantum dots@cellulose nanofiber-clay hybrids for white-light emitting diodes. *J. Mater. Chem. C* **3**, 3536–3541 (2015).
25. Zhang, X. et al. Color-Switchable Electroluminescence of Carbon Dot Light-Emitting Diodes. *Acs Nano* **7**, 11234–11241 (2013).
26. Li, X.M., Rui, M.C., Song, J.Z., Shen, Z.H. & Zeng, H.B. Carbon and Graphene Quantum Dots for Optoelectronic and Energy Devices: A Review. *Adv. Funct. Mater.* **25**, 4929–4947 (2015).
27. Oviedo, J., San-Miguel, M.A., Heredia-Guerrero, J.A. & Benitez, J.J. Electrostatic Induced Molecular Tilting in Self-Assembled Monolayers of n-Octadecylamine on Mica. *J. Phys. Chem. C* **116**, 7099–7105 (2012).
28. Friedel, B. et al. Effects of Layer Thickness and Annealing of PEDOT:PSS Layers in Organic Photodetectors. *Macromolecules* **42**, 6741–6747 (2009).
29. Zhou, J. et al. The temperature-dependent microstructure of PEDOT/PSS films: insights from morphological, mechanical and electrical analyses. *J. Mater. Chem. C* **2**, 9903–9910 (2014).
30. Chu, J.Y.C. & Stolka, M. Thermal-degradation of poly-N-vinylcarbazole. *J. Polym. Sci. Pol. Chem.* **13**, 2867–2870 (1975).
